# Effect of Methadone Maintenance Therapy on Anthropometric Indices in Opioid Dependent Patients

**DOI:** 10.5812/ijhrba.4968

**Published:** 2012-11-26

**Authors:** Farzaneh Montazerifar, Mansour Karajibani, Kobra Lashkaripour

**Affiliations:** 1 Pregnancy Health Research Center, Zahedan University of Medical Sciences, Zahedan, IR Iran; 2 Health Promotion Research Center Zahedan University of Medical Sciences, Zahedan, IR Iran; 3 Department of Nutrition, School of Medicine, Zahedan University of Medical Sciences, Zahedan, IR Iran; 4 Department of Psychiatry ,Baharan Psychiatric Hospital, Zahedan University of Medical Sciences, Zahedan, IR Iran

**Keywords:** Body Mass Index, Population Characteristics, Opioid

## Abstract

**Background:**

Opium abuse significantly affects the nutritional status of users and frequently leads to undernourishment. Methadone maintenance therapy has been used as one of the possible ways to prevent of infection diseases such as HIV and hepatitis B and C and improve the quality of life in opioid-dependent patients.

**Objectives:**

The aim of this study was to assess the anthropometric and socio-demographic characteristics of opium addicted persons before and after 8 weeks of methadone maintenance therapy (MMT).

**Patients and Methods:**

A clinical cross-sectional study was carried out on 55 opium users (15 women and 40 men; mean aged 31.6 ± 10 years), dependent on opium and its derivatives at the Addiction Treatment Clinic of the Baharan psychiatric Hospital, Zahedan, Sistan and Baluchistan Province, Iran, in 2011. The patients were examined before and after 8 weeks MMT. Weight and height of participants were taken and the body mass index (BMI) was calculated.

**Results:**

Body weight increased significantly from 61.4 ± 14.4 to 65.3 ± 14.2 kg and BMI from 21.4 ± 4.2 to 23 ± 5.6 (kg/m^2^) after 8 weeks of methadone maintenance therapy in opium users (P < 0.01). The percentages of underweight, overweight and obese patients were; 27.3%, 18.2% and 3.6%, respectively pre-MMT, and 12.7%, 18.2% and 7.2%, respectively after MMT.

**Conclusions:**

The study shows that methadone Maintenance Therapy led to improvements in nutritional status.

## 1. Background

Opiate abuse is one of the most important social–economical problems around the world. The incidence of which is increasing with the rapid growth of cities and migration. It significantly affects the nutritional and metabolic status of users and frequently leads to undernourishment (1-3). Several studies have reported that drug users demonstrate nutritional deficiencies and changes in their food intake (4, 5). In the short-term Opioid use, heroin in particular, leads to; a dry mouth, and a heavy feeling in the extremities, which may be accompanied by nausea, vomiting, and severe itching, all of these symptoms can affect the individual nutritionally. In the long term it causes; weight loss, malnutrition, and infectious complications (colds, pneumonia and tuberculosis), as a result of secondary immune dysfunction and metabolic disorders, which are all influenced by food intake and/or nutritional status (6). Malnutrition among drug addicts varies from 5% to 30% (5). In nutritional evaluations, anthropometry is an inexpensive technique for assessing the size, proportion and composition of the human body. It reflects both health and nutritional status and predicts performance, health and survival. As such, it is a valuable tool for guiding public health policy and clinical decision making (7). Nutritional screening tools commonly include the body mass index (BMI) as the ‘gold standard’ indicator of malnutrition (8, 9). Moreover, it has been reported that methadone maintenance therapy (MMT) is useful in the treatment of opioid dependence. It has many of the same effects as other opioids including heroin and morphine, with a longer duration of effect (10). MMT has been used in a number of countries for more than 35 years, and it was started in Iran in 1997 (8). Currently, MMT is administered in a wide range of locations in Iran and it allows patients to improve their health and social efficiency (10). Addicts are at risk of malnutrition, and there is currently little data about nutritional status in these patients during methadone maintenance therapy.

## 2. Objectives

The aim of this study was to evaluate the anthropometric and socio-demographic characteristics of opiate-dependent patients undergoing methadone maintenance therapy.

## 3. Patients and Methods

A clinical cross-sectional study was carried out on 55 patients, 15 women and 40 men (mean age 31.6 ± 10 years), range 18 - 67 years, who were Opioid dependent at the Addiction Treatment Clinic of the Baharan psychatric Hospital, Zahedan, Sistan and Baluchestan Province, Iran, in 2011. The anthropometric indices were measured before and after 8 weeks MMT. Measurements of body weight and height, and calculation of BMI were performed for anthropometric evaluation. The participants were weighed with digital scales (Seca, Germany) to the nearest 0.5 kg. Their height was measured with a non-stretch tape fixed to a flat vertical wall, while the patients were without shoes and wearing light clothes. BMI was calculated as weight (kg)/height (m^2^). The standard categorization of BMI was as follows:

(underweight); 18.5 - 24.9 (normal range); 25 - 29.9 (overweight) and ≥ 30 Kg/m^2^ (obese) (9, 11, 12). The protocol of this study was approved by the Ethical Committee of the Zahedan University of Medical Sciences. Prior to the study, subjects were informed about the objectives and methods. All subjects were checked for adverse effects of peripheral edema by a psychiatrist during the follow-up period.

### 3.1. Statistical Analysis

Statistical analysis was performed using SPSS 17 software. The results were represented as mean ± SD and frequency. Differences between the subjects were assessed using non-parametric statistics (independent sample t-test and paired t-test). Pearson’s correlation coefficient was used to determine the relationship between the variables. P < 0.05 was considered as statistical significant.

## 4. Results

The characteristics of the subjects are presented in Table 1. The duration of addiction was 3-50 months. During the study period, eight patients withdrew from the study, the rest of the patients completed the study without any problems.

**Table 1.** Socio-Demographic Characteristics of the Participants

	Drug Addicts (n = 55)
**Gender, No. (%)**	
Male	40 (72.7)
Female	15 (27.3)
Age, y, mean ± SD	31.6 ± 10
Addiction duration, months	3 - 50
**Blood pressure, mmHg, mean ± SD**	* *
Systolic	10.6 ± 0.8
Diastolic	7.2 ± 0.7
**Marital status, No. (%)**	* *
Single	16 (29.1)
Married	37 (67.3)
Divorced/ widow	2 (3.6)
**Employment status, No. (%) ^a^**	* *
Employed	4 (7.4)
Unemployed	10 (18.2)
Laborer	3 (5.6)
Flexible	16 (29.6)
Driver	7 (12.7)
**Education status, No. (%)**	
No education	5 (9.1)
Education	50 (89.9)

^a^All of the women were housekeeper

weeks of methadone maintenance therapy showed a noteworthy increase in body weight (P < 0.01). Categorized BMI values showed a significant difference in the underweight and normal-weight categories, but not in the overweight and obese categories. The majority of subjects had a normal BMI (n = 28; 50.9% vs. n = 34; 61.9%), followed by the overweight (n = 10; 18.2%), underweight (n = 15; 27.3% vs. n = 7; 12.7%) and finally obese participants (n = 2; 3.6% vs. n = 4; 7.3%) (Table 2).

**Table 2.** Anthropometric Characteristics of Study Subjects Before and After MMT

	Drug Abusers	
	Pre-MMT (n = 55)	Post-MMT (n = 47)
**Weight, kg, Mean ± SD**	61.4 ± 14.4	65.3 ± 14.2 ^a^
**BMI, kg/m^2^, Mean ± SD**	21.4 ± 4.2	23 ± 5.6 ^a^
**Underweight, No. (%)**	15 (27.3)	7 (12.7)
**Normal weight, No. (%)**	28 (50.9)	34 (61.9)
**Overweight, No. (%)**	10 (18.2)	10 (18.2)
**Obese, No. (%)**	2 (3.6)	4 (7.3)

Abbreviations: MMT, methadone maintenance therapy

^a^*P* < 0.01 vs. Pre-MMT

The mean weight and BMI levels in educated addicts, who lived with a partner, were higher than those of the other patients (Table 3).

**Table 3.** Social and Anthropometric Characteristics of Participants Before and After MMT

	Weight, kg		BMI, kg/m^2^	
	Pre-MMT (n = 55)	Post-MMT (n = 47)	Pre-MMT (n = 55)	Post-MMT (n = 47)
**Single**	** **	** **	** **	** **
No education (n = 14)	62.6 ± 14.1	63.8 ± 14.1	21.4 ± 4.8	22.5 ± 5.1
Educated (n = 4)	66 ± 14.3	68.2 ± 14.4	21.8 ± 3.8	22.9 ± 4.5
**Married**				
No education (n = 30)	59.6 ± 13.6	62.2 ± 14	20.4 ± 4.6	21.5 ± 4.2
Educated (n = 5)	67.8 ± 13.5	73.8 ± 17.7 ^a^	23.6 ± 4.5	25.9 ± 6.4 ^a^
**Divorced/ widow**				
No education (n = 2)	63.5 ± 10.6	56 ± 9.5	23.2 ± 2.1	23.3 ± 2.2
Educated (n = 0)	-	-	-	-

Abbreviations: MMT, methadone maintenance therapy; NS, not significant

^a^*P* < 0.01 vs. Pre-MMT

## 5. Discussion

In this present study, we examined the BMI (as a criterion for assessing under-nutrition) (9), and socio-demographic characteristics of the patients before and after 8 weeks of MMT. The results showed that the majority of patients had achieved an appropriate nutritional status evaluated by their BMI, before treatment only 50.9% had a normal BMI at baseline. This is consistent with previous studies (13-15). The prevalence of underweight patients (BMI < 18.5 kg/m^2^) was found more often than overweight (BMI 25 - 29.9 kg/m^2^), and obesity (BMI ≥ 30 kg/m^2^), among the examined patients. During the 8 weeks of MMT, a significant increase in BMI was observed. More of the patients had a normal BMI, particularly in the male group. The same was true for body weight which increased significantly, and this result is similar to several previous studies (16-18), but unlike the Forrester et al. study (19). The findings of our study demonstrate that MMT improves nutritional status. Weight gain in methadone-maintenance patients has been documented in previous studies, and this may be derived from increasing fat body mass (14, 18). It might also partly be due to adipose tissue-derived hormones, such as leptin, which are related to body fat content (1). The relationship between education level, employment, marital status and BMI has been reported in earlier studies (2, 15), but our study differed from the results of Saeland et al. (5). In our study, the mean levels of weight and BMI were higher in educated subjects who lived with a partner, compared to the others, while employment status showed no significant difference between the two groups (data not shown). Usually, the presence of someone in the house who prepares meals seems to help the patient’s motivation to eat. So, healthcare professionals should pay more attention to the social problems of these patients (1). Although there was some evidence concerning the association between anthropometric and socio-demographic characteristics with drug use and MMT, so far only a little data has been made available on the particular factors of MMT. Thus, future studies will be necessary to assess the impact of other factors, such as food behavior, dietary intake, carbohydrate craving, exercise, adipocyte-derived hormones and non-dietary determinants of nutritional status in the opioid-using population. Opioid abuse was associated with increased numbers of underweight patients. Substitution treatment of Opioid dependency with methadone led to improvements in the nutritional status by means of decreasing the numbers who were underweight. Limitations of this study which need to be given more attention in the future include: 1) Longer study duration, this may give a much better therapeutic evaluation of the intervention. 2) Measurement of adipose tissue-derived hormones, which are related to body fat content such; as leptin and adiponectin. 3) Evaluation of nutritional status based on nutrient intake and, 4) the selection of a control group.
